# Comparison of the Sulfonamide Inhibition Profiles of the α-Carbonic Anhydrase Isoforms (SpiCA1, SpiCA2 and SpiCA3) Encoded by the Genome of the Scleractinian Coral *Stylophora pistillata*

**DOI:** 10.3390/md17030146

**Published:** 2019-03-01

**Authors:** Sonia Del Prete, Silvia Bua, Fatmah A. S. Alasmary, Zeid AlOthman, Sylvie Tambutté, Didier Zoccola, Claudiu T. Supuran, Clemente Capasso

**Affiliations:** 1 Istituto di Bioscienze e Biorisorse, National Research Council (CNR), Via Pietro Castellino 111, 80131 Napoli, Italy; sonia.delprete@ibbr.cnr.it; 2 Dipartimento Neurofarba, Sezione di Scienze Farmaceutiche e Nutraceutiche, Università degli Studi di Firenze, Via U. Schiff 6, 50019 Sesto Fiorentino, Florence, Italy; silvia.bua@unifi.it (S.B.); claudiu.supuran@unifi.it (C.T.S.); 3 Department of Chemistry, College of Science, King Saud University, P.O. Box 2455 Riyadh 11451, Saudi Arabia; fasmari@ksu.edu.sa (F.A.S.A.); zaothman@KSU.EDU.SA (Z.A.); 4 Department of Marine Biology, Centre Scientifique de Monaco, 8 Quai Antoine 1, 98000 Monaco, Monaco; stambutte@centrescientifique.mc

**Keywords:** carbonic anhydrases, sulfonamides, CA isoforms, biomineralization, corals CAs, recombinant enzyme

## Abstract

The ubiquitous metalloenzymes carbonic anhydrases (CAs, EC 4.2.1.1) are responsible for the reversible hydration of CO_2_ to bicarbonate (HCO_3_^−^) and protons (H^+^). Bicarbonate may subsequently generate carbonate used in many functional activities by marine organisms. CAs play a crucial role in several physiological processes, e.g., respiration, inorganic carbon transport, intra and extra-cellular pH regulation, and bio-mineralization. Multiple transcript variants and protein isoforms exist in the organisms. Recently, 16 α-CA isoforms have been identified in the coral *Stylophora pistillata*. Here, we focalized the interest on three coral isoforms: SpiCA1 and SpiCA2, localized in the coral-calcifying cells; and SpiCA3, expressed in the cytoplasm of the coral cell layers. The three recombinant enzymes were heterologously expressed and investigated for their inhibition profiles with sulfonamides and sulfamates. The three coral CA isoforms differ significantly in their susceptibility to inhibition with sulfonamides. This study provides new insights into the coral physiology and the comprehension of molecular mechanisms involved in the bio-mineralization processes, since CAs interact with bicarbonate transporters, accelerating the trans-membrane bicarbonate movement and modulating the pH at both sides of the plasma membranes.

## 1. Introduction

Coral reef architecture comes from the deposition of massive calcium carbonate skeletons secreted by scleractinian corals, or hard corals [1]. These corals represent the habitat for a vast diversity of organisms [2]. They live in intimate symbiosis with unicellular dinoflagellate symbionts, commonly called zooxanthellae, which are hosted in the coral tissues. The enzymes carbonic anhydrases (CAs, EC 4.2.1.1) play major roles in two essential processes of coral’s physiology; they are involved in the carbon supply for calcium carbonate precipitation (formation of skeletons) as well as in carbon-concentrating mechanisms for symbiont photosynthesis [3]. Numerous studies have shown CAs are ubiquitous metallo-enzymes, which are responsible for the reversible hydration of CO_2_ to bicarbonate (HCO_3_^−^), carbonate (CO_3_^2−^), and protons (H^+^) [2,4,5,6,7,8,9,10,11,12,13,14,15]. In the biological systems, the CO_2_, HCO_3_^−^, CO_3_^2−^, and H^+^ are interconnected by equilibrium reactions, and their concentrations are regulated by CAs [5,16,17,18,19]. Thus, these enzymes play a crucial role in several physiological processes such as respiration, inorganic carbon transport, intra and extra-cellular pH regulation, or the bio-mineralization process [20,21,22,23,24]. Up to the present time, seven polyphyletic classes of CAs have been described and indicated with the Greek letters α, β, γ, δ, ζ, η and θ [19,25,26,27]. Moreover, members of each class possess multiple transcript variants and protein isoforms, which are characterized by different biochemical properties and have specific tissue/organ and sub-cellular localizations [4,5,17,28,29,30,31]. For examples, CAs present in animals belong to α-class [32,33], plants and algae have α-, β-, γ-, δ- and θ-classes; fungi encode for α- and β-CAs; protozoa for α-, β- and/or η-CAs; bacteria for α-, β- and γ-CA classes [30,31,34,35,36,37,38]. Intriguing, in the oyster *Pinctada fucata* a matrix protein, called nacrein, has been identified. It participates in the formation of the nacreous layer and is characterized by a CA domain present at the N-terminus part of the polypeptide sequence [39]. In mammals, 16 α-CA isoforms have been identified: Eight of them are cytosolic, five are membrane-bound, two are mitochondrial, and only one is secreted, the last three being devoid of catalytic activity and referred to as CA Related Proteins (CARPs) [16,18,27]. 

In corals, most of the available results on CAs were obtained by measuring the CA activity in crude tissue extracts using non-specific CA inhibitors or antibodies raised against human isoforms [6,7,14,40,41]. Recently, the development of molecular biology tools allowed the isolation and full characterization of several CA isoforms in different coral species, such as *Lobactis scutaria* [42], *Stylophora pistillata* [2,8], and *Acropora millepora* [43]. In particular, our groups analyzing the molecular data in the branching coral *Stylophora pistillata* identified 16 α-CA isoforms in the transcriptome and genome of this scleractinian coral [2,3,8,44,45,46,47,48]. Among them, two α-CAs were isolated (STPCA and STPCA-2, here, indicated as SpiCA1 and SpiCA2, respectively) and have been localized in the coral-calcifying cells, within the epithelium facing the skeleton [2,8]. It has been proposed that SpiCA1 catalyzes the inter-conversion between the different inorganic forms of dissolved inorganic carbon at the site of calcification, whereas SpiCA2 is an intracellular enzyme, which is found as an organic matrix protein incorporated in the skeleton [49,50]. Recently, a novel α-CA, named SpiCA3, which is cytoplasmic and ubiquitously expressed in all the coral cell layers, has been characterized [20]. This isoform showed a catalytic activity 1.14-times higher than human CA II and is one of the most effective CO_2_ catalysts among all CAs known to date with a k*_cat_* of 1.6 × 10^6^ s^−1^ and a k*_cat_*/K_M_ of 1.5 × 10^8^ M^−1^ s^−1^ [20]. Intriguingly, the three coral CAs (SpiCA1, SpiCA2, and SpiCA3) differ significantly in their catalytic activity and susceptibility to inhibition with anions [20]. Here, the sulfonamide inhibition profile of the SpiCA3 has been investigated for the first time. Sulfonamides and their bio-isosteres represent the most important class of CA inhibitors (CAIs). Furthermore, the SpiCA3 inhibition profile was compared with those obtained for SpiCA1 and SpiCA2 previously studied by our groups [45].

## 2. Results and Discussion

### 2.1. Recombinant Enzymes

**Figure 1:**
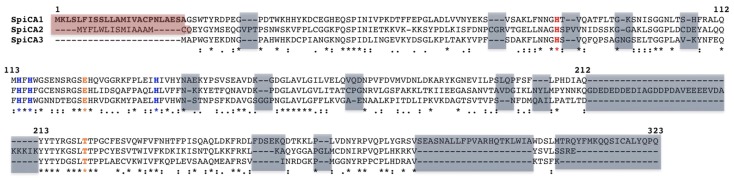
This Multiple amino acid sequence alignment of the α-CAs encoded by the genome of *S. pistillata* (SpiCA1, SpiCA2, and SpiCA3). The main features of α-CA are indicated with different colors: zinc ligands are in blue; the “gate-keeper” residues are in orange; the histidine proton shuttle is in red; long stretches of 31 and 35 amino acid residues, in black bold. The insertion or deletion of amino acid residues are indicated with the grey transparent boxes, while the signal peptides typifying the SpiCA1 and SpiCA2 isoforms are included in the red transparent box. The symbol (*) signifies identity at a position, while the symbols (:) and (.) designates conserved and semi-conserved substitutions, respectively. The SpiCA1 numbering system was used. The multiple sequence alignment was performed with the program Muscle Ver. 3.8. SpiCA1, *S. pistillata* isoform 1 (accession no. ACA53457.1); SpiCA2, *S. pistillata* isoform 2 (accession no. EU532164.1); and SpiCA3, *S. pistillata* isoform 3 (accession no. XP_022794253.1).

The recombinant SpiCA1, SpiCA2, and SpiCA3 were obtained, as described earlier [2,20,44,51,52]. Figure 1 shows a multi-alignment of the three α-CA isoforms encoded by the genome *S. pistillata* and investigated up until now. It is readily apparent that the three coral isoforms show the main features of a typical mammalian α-CAs. They possess the conserved: (i) Three His ligands, which coordinate the Zn(II) ion crucial for catalysis, (His94, His96, and His119, hCA I numbering system); (ii) the two gate-keeping residues (Glu106 and Thr199), which are implicated in the substrate orientation and the binding of the inhibitors; and (iii) the proton shuttle residue (His64), which is involved in the transfer of the proton (H^+^) from the water coordinated to the Zn(II) ion to the environment, influencing and making very fast the rate of the catalytic reaction. Furthermore, SpiCA3, diversely from the other two coral isoforms, is a cytoplasmic protein. SpiCA1 and SpiCA2 are, in fact, secreted proteins characterized by the presence of a signal peptide at the N-terminal of their amino acid sequences (see Figure 1). Interesting, the insertions and deletions of a relatively extended number of amino acid residues along the polypeptide chain, which affect the three coral isoforms (Figure 1), may influence the kinetic and inhibition behavior of the coral enzymes, probably because of significant alterations of their three-dimensional structure. For example, SpiCA3 showed a k*_cat_* = 10^6^ s^−1^, which is one order of magnitude higher than the k*_cat_* (10^5^ s^−1^) of the other two isoforms. 

### 2.2. Sulfonamide Used as CAIs

**Figure 2:**
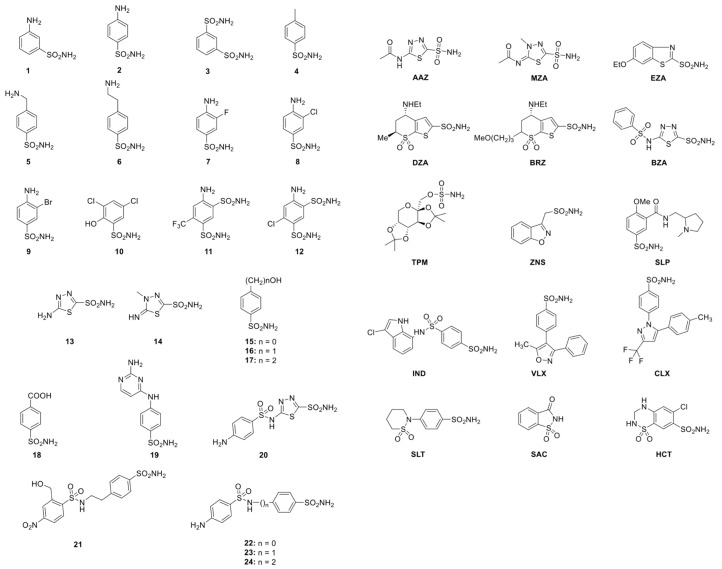
38 sulfonamides and one sulfamate (Topiramate (TPM)) used for studying the inhibition profile of the three coral isoforms (SpiCA1, SpiCA2, and SpiCA3).

As described in the literature, it has been demonstrated that the sulfonamide CA inhibitors (CAIs), such as acetazolamide or ethoxzolamide, drastically decrease the coral calcification rates, with inhibition of up to 73% [50]. These data suggest that the coral CAs are finely tuned in providing carbonate and H^+^ ions for the control of the calcification process and pH homeostasis, respectively [6,14,53,54]. Unfortunately, very few studies are available on the inhibition of the CAs encoded by coral genomes. The CAIs can be clustered into several different groups considering their binding mode to the enzyme active site [29,55]: (1) The metal ion binders (anion, sulfonamides and their bioisosteres, dithiocarbamates, xanthates, etc.); (2) compounds which anchor to the zinc-coordinated water molecule/hydroxide ion (phenols, polyamines, thioxocoumarins, sulfocumarins); (3) compounds occluding the active site entrance, such as coumarins and their isosteres; (4) compounds binding out of the active site, such as an aromatic carboxylic acid derivative; and (5) inhibitors with an unknown binding mechanism, such as secondary/tertiary sulfonamides, protein tyrosine kinase inhibitors, and fullerenes, for which the X-ray crystallographic structure is unavailable [29]. The most investigated CAIs are the anions and the sulfonamides [19,29,56,57]. A library of 40 compounds, 39 primary sulfonamides, and one sulfamate, were used as CAIs (Figure 2). Derivatives **1–24** and **AAZ-HCT** are either simple aromatic/heterocyclic sulfonamides widely used as building blocks for obtaining new families of such pharmacological agents, or they are clinically used agents, among which acetazolamide (**AAZ**), methazolamide (**MZA**), ethoxzolamide (**EZA**), and dichlorophenamide (**DCP**) are the classical, systemically acting antiglaucoma CAIs. Dorzolamide (**DZA**) and brinzolamide (**BRZ**) are topically acting antiglaucoma agents; benzolamide (**BZA**) is an orphan drug belonging to this class of pharmacological agents.

**Figure 3:**
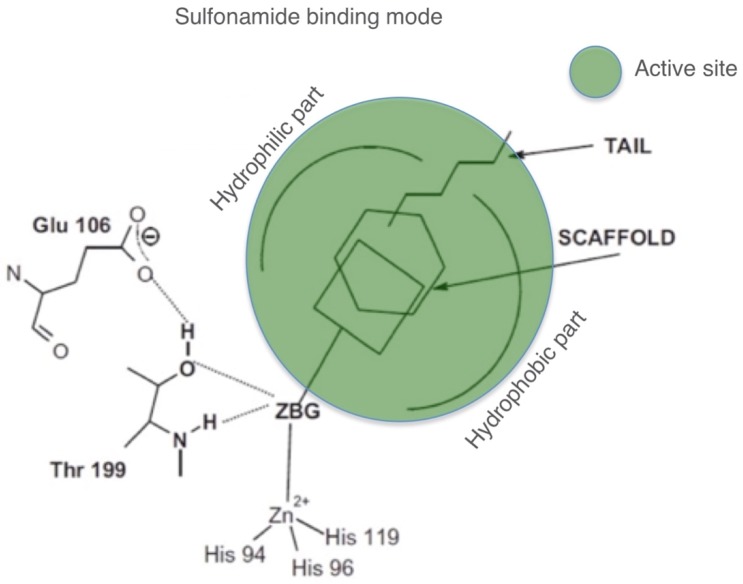
Schematic representation of the metal binding mode of CAIs to human (h) CA isoform hCA II, such as sulfonamides and their bio-isosters.

Moreover, the zonisamide (**ZNS**), sulthiame (**SLT**), and the sulfamic acid ester topiramate (**TPM**) are widely used antiepileptic drugs. Sulpiride (**SLP**) and indisulam (**IND**) were also shown by our group to belong to this class of pharmacological agents, together with the COX2 selective inhibitors celecoxib (**CLX**) and valdecoxib (**VLX**). Saccharin (**SAC**) and the diuretic hydrochlorothiazide (**HCT**) are also known to act as CAIs. As shown in Figure 3, sulfonamides, such as the clinically used derivatives acetazolamide, methazolamide, ethoxzolamide, dichlorophenamide, dorzolamide, and brinzolamide, bind in a tetrahedral geometry to the Zn(II) ion in the deprotonated state, with the nitrogen atom of the sulfonamide moiety coordinated to Zn(II) and an extended network of hydrogen bonds, involving amino acid residues of the enzyme, also participating in the anchoring of the inhibitor molecule to the metal ion [19,29,55,58]. The aromatic/heterocyclic part of the inhibitor interacts with the hydrophilic and hydrophobic residues of the catalytic cavity (Figure 3) [19,29,56,57]. 

### 2.3. CA Inhibition Data and Comparative Analysis

Table 1 shows inhibition data of sulfonamides (and one sulfamate, TPM) against the human α-CAs (isoforms hCA I and hCA II) and the recombinant coral α-CA isoforms (SpiCA1, SpiCA2, and SpiCA3). Recently, our groups reported data for hCAI, hCAII, SpiCA1, and SpiCA2 earlier [8,45]. The following should be noted regarding the inhibition of the three coral enzymes with the compounds investigated in this study:

*(i) High potency inhibitors*

Most of the tested sulfonamides were effective inhibitors of the coral isoform SpiCA1 with a K_i_ in the range of 16–92 nM. This is the case of the compounds **5**, **7**, **8**, **14**, **18**, **19**, **20**, **AAZ**, **MZA**, **EZA**, **DZA**, **BRZ**, **BZA**, **TMP**, **VLX**, **CLX**, **SLT**, and **SAC**. Intriguingly, the majority of these compounds were moderate inhibitors of SpiCA2 and SpiCA3 showing a K_I_ > 100 nM. The SpiCA2 inhibition profile showed only one compound (**AAZ**) with a K_I_ < 100 nM; while SpiCA3 was well inhibited by compounds **17**, **19**, **20**, **21**, **23**, **24**, and **IND**. 

*(ii) Medium potency inhibitors*

A large number of simple aromatic sulfonamides, such as derivatives **2–20**, and the pharmacological sulfonamides **AAZ**, **MZA**, **EZA**, **DZA**, **BRZ**, **BZA**, **ZNS**, **TMP**, **SLP**, **IND**, **CLX**, **SLT**, and **SAC** showed moderate SpiCA2 inhibitory properties with a K_I_ in the range 105–868 nM. Intriguing, the sulfonamide inhibition profile of SpiCA2 was characterized mainly by moderate inhibitors (Table 1). The compounds, which resulted in effective and moderate inhibitors of SpiCA1 or moderate inhibitors of SpiCA2, such as **2**, **5**, **6**, **7**, **8**, **TMP**, **ZNS**, **SLP**, **CLX**, and **SAC** resulted in the worst inhibitors for the coral isoform SpiCA3. The majority of these derivatives are benzenesulfonamides with one or two simple substituents in ortho, para, or the 3,4-positions of the aromatic ring with respect to the sulfamoyl zinc-binding moiety.

*(iii) Ineffective inhibitors*

A large number of sulfonamides, such as derivatives **1**, **2**, **5**, **6**, **7**, **8**, **10**, **TPM**, **ZNS**, **SLP**, **VLX**, **CLX**, and **SAC** were weak inhibitors of SpiCA3 showing a K_i_ > 1000 nM. Interesting, the coral isoform SpiCA2 showed only one ineffective derivative (**VLX**), while the SpiCA1 was the isoform better inhibited by all the compounds used in the present study.

*(iv) Human isoforms versus coral enzymes*

The comparison of the inhibition profile of the human isoforms with those of the coral enzymes showed that SpiCA1 resulted very similar to the isoform hCAII. Furthermore, the isoform hCAI was not inhibited by most of the derivatives indicated with the numbers **1**, **2,** and those of the range **4–17** (K_I_ > 1000 nM). Moreover, the clinically used agents, among which DZA, BRZ, and CLX didn’t affect the hCAI activity, while they were high potency inhibitors for hCAII and SpiCA1 and low potency inhibitors for SpCA2 and SpiCA3. 

## 3. Material and Methods

### 3.1. Isoform Expression and Purification

The recombinant SpiCA1, SpiCA2, and SpiCA3 were obtained, as described earlier [2,20,44,51,52]. Briefly, the BL21 DE3 competent cells (Agilent) were transformed with the recombinant vectors containing one of the three coral isoforms, grown at 37 °C and induced with 0.1 mM IPTG. After 30 min, ZnSO_4_ (0.5 mM) was added to the culture medium and cells were grown for an additional four h. Then, cells were harvested and re-suspended in the following buffer: 50 mM Tris/HCl, pH 8.0, 0.5 mM PMSF, and 1 mM benzamidine. Subsequently, bacterial cells containing the overexpressed CAs were disrupted by sonication at 4 °C and centrifugated at 12,000× *g* for 45 min. The resultant supernatant was loaded onto a His-select HF Nickel affinity column (GE Healthcare, dimension: 1.0 × 10.0 cm), equilibrated with 0.02 M phosphate buffer (pH 8.0) containing 0.01 M imidazole and 0.5 M KCl at a flow rate of 1.0 mL/min. The recombinant proteins were eluted from the column using 0.3 M imidazole, and then, dialyzed against 50 mM Tris/HCl, pH 8.3. 

### 3.2. Amino Acid Sequence Analysis

Multi-alignment of amino acid sequences was performed using the program MUSCLE (MUltiple Sequence Comparison by Log-Expectation, EMBL-EBI in Hinxton, Cambridge (UK) Version 3.8), a new computer program for creating multiple alignments of protein sequence [59].

### 3.3. Enzyme Inhibition Profile

An Applied Photophysics stopped-flow instrument (Leatherhead, Surrey (UK)) has been used for assaying the CA catalyzed CO_2_ hydration activity [60]. Phenol red (at a concentration of 0.2 mM) has been used as an indicator, working at the absorbance maximum of 557 nm, with 20 mM TRIS (pH 8.3) as buffer, and 20 mM NaClO_4_ (for maintaining constant the ionic strength), following the initial rates of the CA-catalyzed CO_2_ hydration reaction for a period of 10–100 s. The CO_2_ concentrations ranged from 1.7 to 17 mM for the determination of the kinetic parameters (by Lineweaver-Burk plots) and inhibition constants. For each inhibitor, at least six traces of the initial 5%–10% of the reaction have been used for determining the initial velocity. The un-catalyzed rates were determined in the same manner and subtracted from the total observed rates. Stock solutions of inhibitor (10–100 mM) were prepared in distilled-deionized water, and dilutions up to 0.01 mM were done after that with the assay buffer. Inhibitor and enzyme solutions were preincubated together for 15 min at room temperature before assay, to allow for the formation of the E-I complex or the eventual active site-mediated hydrolysis of the inhibitor. The inhibition constants were obtained by non-linear least-squares methods using PRISM 3 and the Cheng-Prusoff equation, as reported earlier [61,62,63], and represent the mean from at least three different determinations. All CA isoforms were recombinant ones obtained in-house. All salts/small molecules were of the highest purity available, from Sigma-Aldrich (Milan, Italy). 

## 4. Conclusions

In general, CAs are metalloenzymes integrated with a structural-functional complex of sequential enzymes [64,65,66] interconnected by metabolites produced from one catalyst and passed into the active site of another enzyme [64,65]. For example, CA isoforms interact with bicarbonate transporters increasing the local bicarbonate concentration, and thus, accelerating the transmembrane bicarbonate movement, and modulating the pH at both sides of the plasma membranes [64,66]. All these metabolic processes influence the coral physiology. Therefore, the study of the coral CA inhibition profiles may provide new insights to design experiments aimed at a better understanding of the molecular mechanisms involved in coral biomineralization and symbiosis. Furthermore, from the Table 1, it is readily apparent that the sulfonamide inhibition profile of the coral isoforms is substantially different from those of the cytosolic human isoforms, proving that it might be possible to design selective inhibitors using the scaffold of leads detected here for producing antiinfectives agents towards the pathogenic CAs [67]. The study of the inhibition profiles of new CAs, such as the coral ones, will give new advances in the synthesis of novel CAIs or in the modification/optimization of the existing inhibitors for making them more selective towards the pathogenic CAs. 

## Figures and Tables

**Table 1:** Inhibition of human α-CAs (hCA I and hCA II) and the three recombinant enzyme from S. pistillata (SpiCA1, SpiCA2, and SpiCA3) with sulfonamides **1–24** and the clinically used drugs AAZ—HCT reported in Figure 2.

Inhibitor		K_I_ * (nM)			
	hCA I ^a^	hCA II ^a^	SpiCA1 ^a^	SpiCA2 ^a^	SpiCA3
**1**	28,000	300	-	-	5059
**2**	25,000	240	364	300	4276
**3**	79	8	-	-	667
**4**	78,500	320	614	516	694
**5**	25,000	170	83	508	7871
**6**	21,000	160	94	577	7828
**7**	8300	60	75	493	3318
**8**	9800	110	88	551	1815
**9**	6500	40	104	540	918
**10**	7300	54	-	-	2532
**11**	5800	63	367	481	856
**12**	8400	75	295	840	430
**13**	8600	60	105	361	275
**14**	9300	19	92	357	578
**15**	5500	80	-	-	487
**16**	9500	94	-	-	199
**17**	21,000	125	770	701	66
**18**	164	46	30	661	241
**19**	109	33	25	868	83
**20**	6	2	28	333	74
**21**	69	11	-	-	53
**22**	164	46	-	-	568
**23**	109	33	-	-	62
**24**	95	30	-	-	46
**AAZ**	250	12	16	74	737
**MZA**	50	14	21	132	821
**EZA**	25	8	39	105	56
**DZA**	50,000	9	18	113	354
**BRZ**	45,000	3	48	169	250
**BZA**	15	9	20	214	394
**TPM**	250	10	29	367	5828
**ZNS**	56	35	259	645	5513
**SLP**	1200	40	430	415	>10,000
**IND**	31	15	163	394	92
**VLX**	54,000	43	29	5710	2918
**CLX**	50,000	21	34	690	9102
**SLT**	374	9	45	123	251
**SAC**	18,540	5959	40	104	>10,000
**HCT**	328	290	-	-	243

* Errors in the range of 5–10% of the reported data, from 3 different assays (data not shown) ^a^ Human recombinant isozymes and coral recombinant isoforms, stopped flow CO_2_ hydrase assay method, from References 12 and 45. - means not tested.
